# Lingual and Cervical Ectopic Thyroid: A Case Report and Literature Review

**DOI:** 10.7759/cureus.101328

**Published:** 2026-01-12

**Authors:** Thamiris C Bená, Rafael G De Medeiros, Yasmin Duran, Antonio V Priante

**Affiliations:** 1 Head and Neck Surgery, Taubate University, Taubaté, BRA; 2 Head and Neck Surgery, Taubate University, Taubate, BRA

**Keywords:** congenital abnormalities, diagnostic imaging, hypothyroidism, lingual thyroid, thyroid dysgenesis, thyroidectomy

## Abstract

Multiple foci of ectopic thyroid tissue are rare and result from abnormal migration of the thyroid gland during embryonic development. This condition may manifest at various sites along the thyroglossal tract. Although often asymptomatic, depending on the location and functional capacity of the ectopic gland, it can cause significant compressive symptoms and may be associated with congenital or acquired hypothyroidism. We reported the case of a 37-year-old woman with hypothyroidism who had a symptomatic cervical nodule with progressive enlargement. Imaging revealed an ectopic thyroid at the base of the tongue and in the cervical region adjacent to the thyroid cartilage, as well as the absence of the thyroid gland in its normal anatomical position. Surgical intervention was indicated for compressive symptoms, and histopathological examination confirmed the diagnosis of ectopic thyroid tissue. Differential diagnosis with other cervical masses is essential, underscoring the importance of embryological and anatomical knowledge of the thyroid gland for appropriate clinical management.

## Introduction

An ectopic thyroid gland is a rare congenital anomaly resulting from a defect in the thyroid gland's migration during embryonic development. Under normal circumstances, the thyroid originates at the foramen cecum at the base of the tongue and migrates to its definitive position in the pretracheal region. When this process is disrupted, thyroid tissue may remain in atypical locations, including the base of the tongue, mediastinum, submandibular region, and along the course of thyroid descent [1, 2].

Clinically, ectopic thyroid tissue may present in multiple ways, depending on its location and functional impact. In many cases, it is asymptomatic and incidentally detected on imaging studies [3, 4, 5]. However, symptoms such as dysphagia, dysphonia, speech changes, including steatomalalia, foreign body sensation, coughing, snoring, and sleep apnea may be found when the gland is located at the base of the tongue. In contrast, the cervical region is frequently asymptomatic; when symptomatic, it may lead to symptoms such as dry cough, dysphagia, and stridor [5, 6]. Additionally, ectopic thyroid tissue may be associated with hormonal dysfunction, including congenital or acquired hypothyroidism, particularly when the ectopic tissue represents the sole source of thyroid hormone production [7].

The diagnosis of ectopic thyroid is established through correlation of clinical, laboratory, and imaging findings [1, 8]. Differential diagnosis with other cervical masses is essential to guide appropriate management, which should be individualized according to symptom severity and associated conditions. The incidence of ectopic thyroid is estimated at approximately 1 in 100,000 individuals. It is more prevalent in women and is often diagnosed during childhood or adolescence, although it may remain asymptomatic throughout life [3, 4]. Therefore, this case report aims to describe the clinical presentation, diagnostic findings, and therapeutic management of a patient with ectopic thyroid tissue in the cervical and lingual regions. Additionally, it seeks to emphasize the importance of imaging and laboratory investigations in the differential diagnosis of cervical lesions and in determining the most appropriate clinical approach.

## Case presentation

A 37-year-old woman with a history of hypothyroidism, currently treated with levothyroxine, and a known thyroid malformation under endocrinological follow-up for seven years, was evaluated. Over the past two years, she has complained of progressive cervical enlargement associated with dysphagia for solid foods and frequent choking episodes, with gradual worsening of symptoms. There was no family history of thyroid dysfunction or malformations.

Physical examination revealed a mobile nodule measuring approximately 3 cm, located at the level of the thyroid cartilage in the right paramedian region, which moved with swallowing.

**Figure 1:**
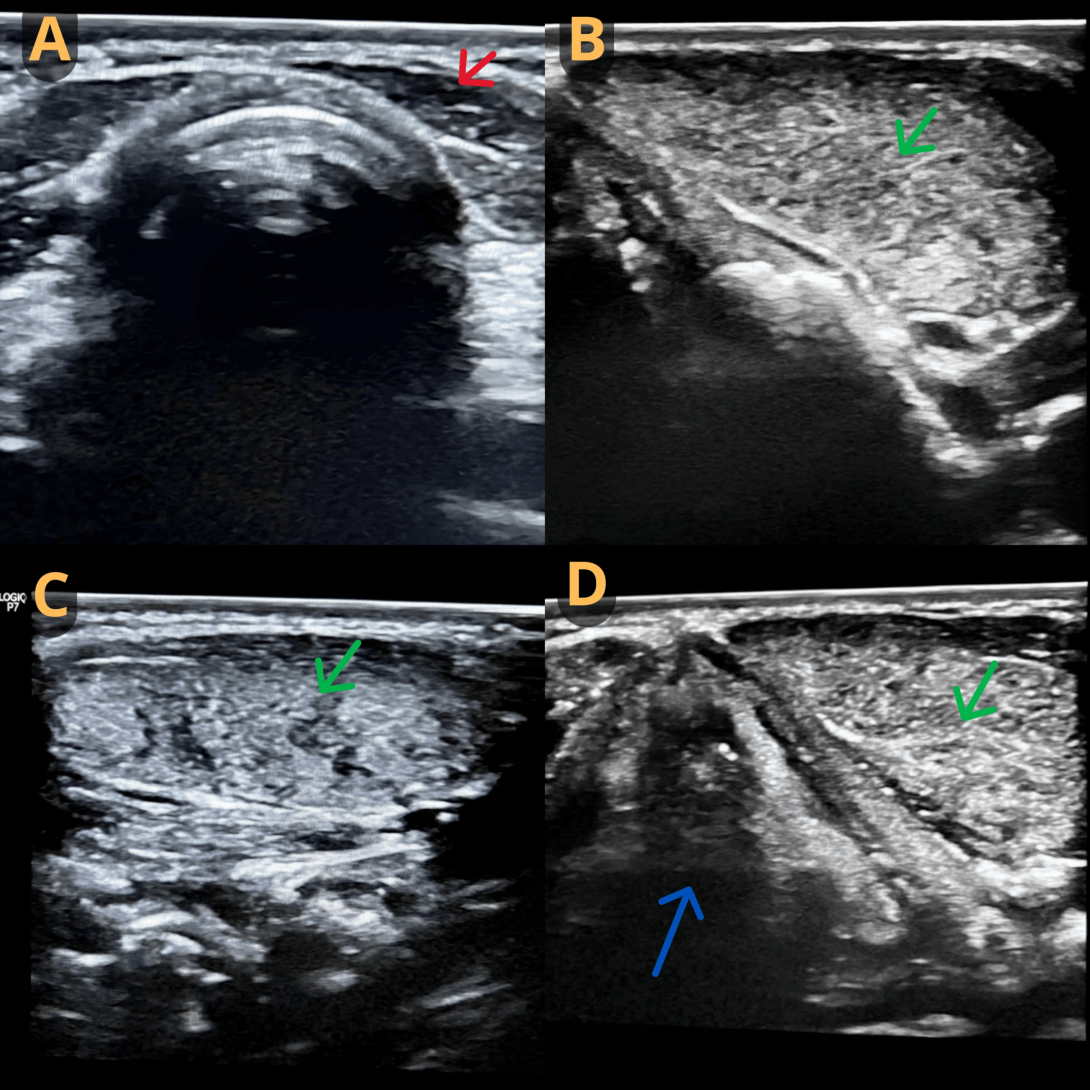
Ultrasonography of the cervical region Absence of an orthotopic thyroid gland (A), hyperechoic nodule in the right paramedian anterior cervical region (B) adjacent to the laryngeal thyroid cartilage (C, D). Red arrow: empty thyroid gland; Green arrow: thyroid parenchyma; Blue arrow: larynx

**Figure 2:**
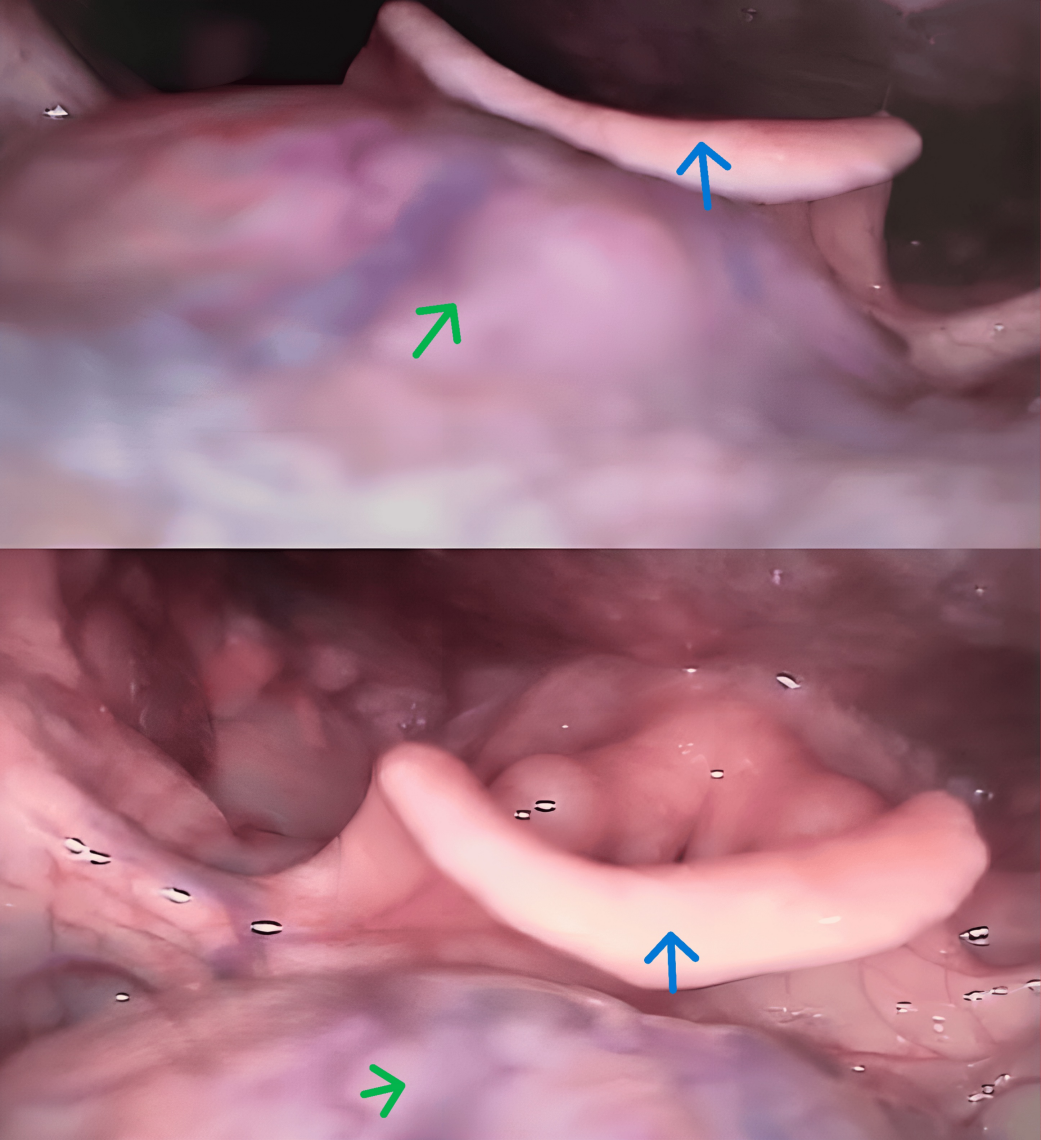
Videolaryngoscopy Nodule at the base of the tongue, consistent with lingual thyroid tissue (green arrow). Blue arrow: epiglottis

Previous ultrasonography (US) demonstrated a hyperechoic nodule in the right paramedian anterior cervical region, measuring 3.0 × 1.0 × 1.5 cm, adjacent to the larynx, as well as the absence of an orthotopic thyroid gland (Figure 1). Fine-needle aspiration biopsy (FNAB) showed no evidence of malignancy. Videolaryngoscopy revealed a submucosal mass of approximately 3.0 cm at the base of the tongue (Figure 2). Based on these findings, ectopic thyroid was suspected, and laboratory tests and computed tomography (CT) were requested.

**Figure 3:**
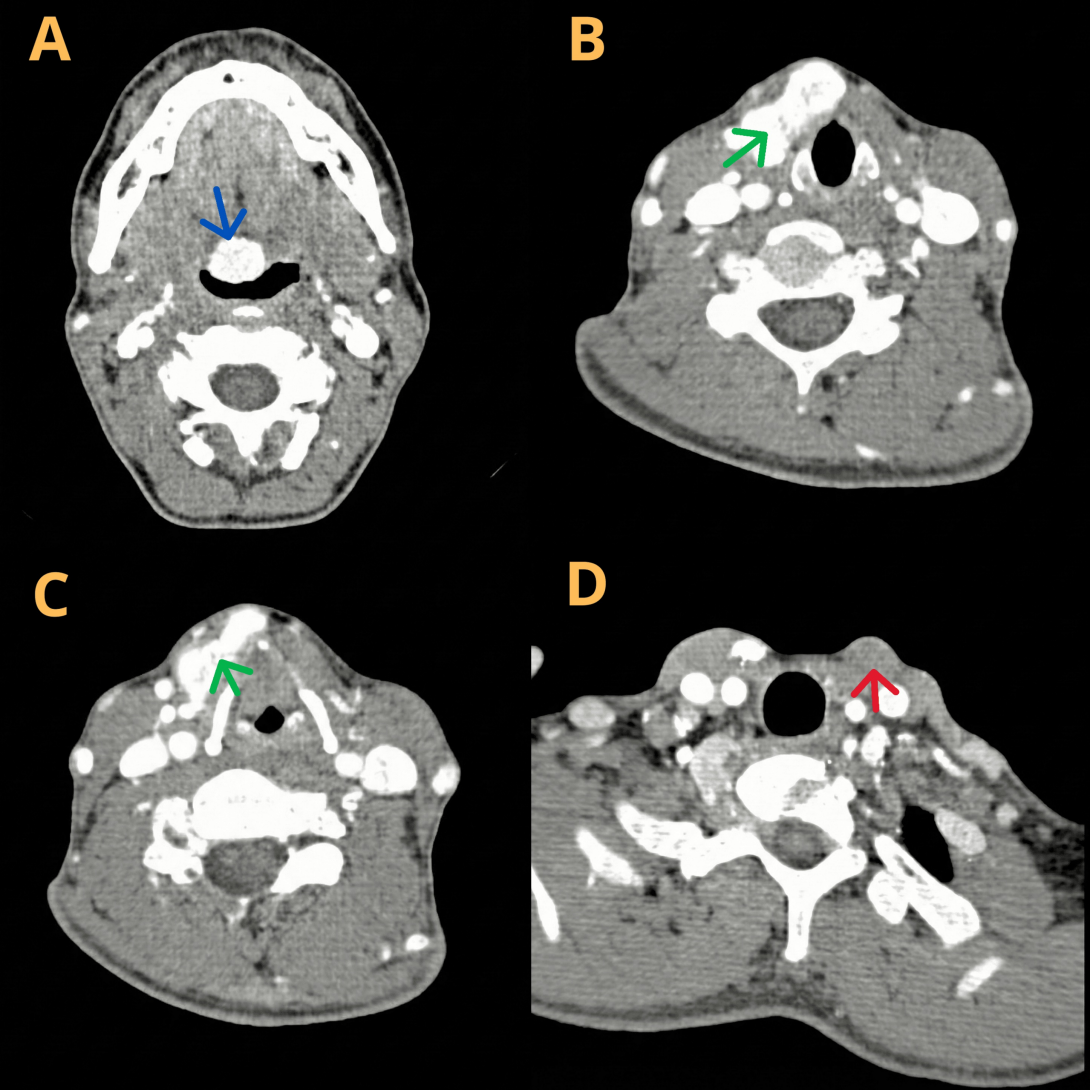
Sagittal computed tomography of the cervical region. Hypervascular expansile lesions at the base of the tongue (A) and in the visceral neck area at the level of the larynx (B, C) and absence of an orthotopic thyroid gland (D). Red arrow: empty thyroid cavity; Green arrow: thyroid parenchyma adjacent to the larynx; Blue arrow: thyroid at the base of the tongue.

Laboratory and imaging findings demonstrated altered thyroid function, with a thyroid-stimulating hormone (TSH) level of 7.58 µIU/mL (reference range: 0.4-4.5 mIU/L) and a free T4 level of 0.87 ng/dL (reference range: 0.8-1.8 ng/dL), in addition to elevated anti-thyroid peroxidase antibodies (146.3 IU/mL) and undetectable anti-thyroglobulin antibodies (<0.9 IU/mL). CT of the cervical region revealed hypervascular expansile lesions located at the base of the tongue, measuring 17 × 13 mm, and in the right anterolateral visceral neck region, measuring 29 × 13 mm (Figure 3). The levothyroxine dose was adjusted, and surgical resection of the cervical lesion was scheduled.

**Figure 4:**
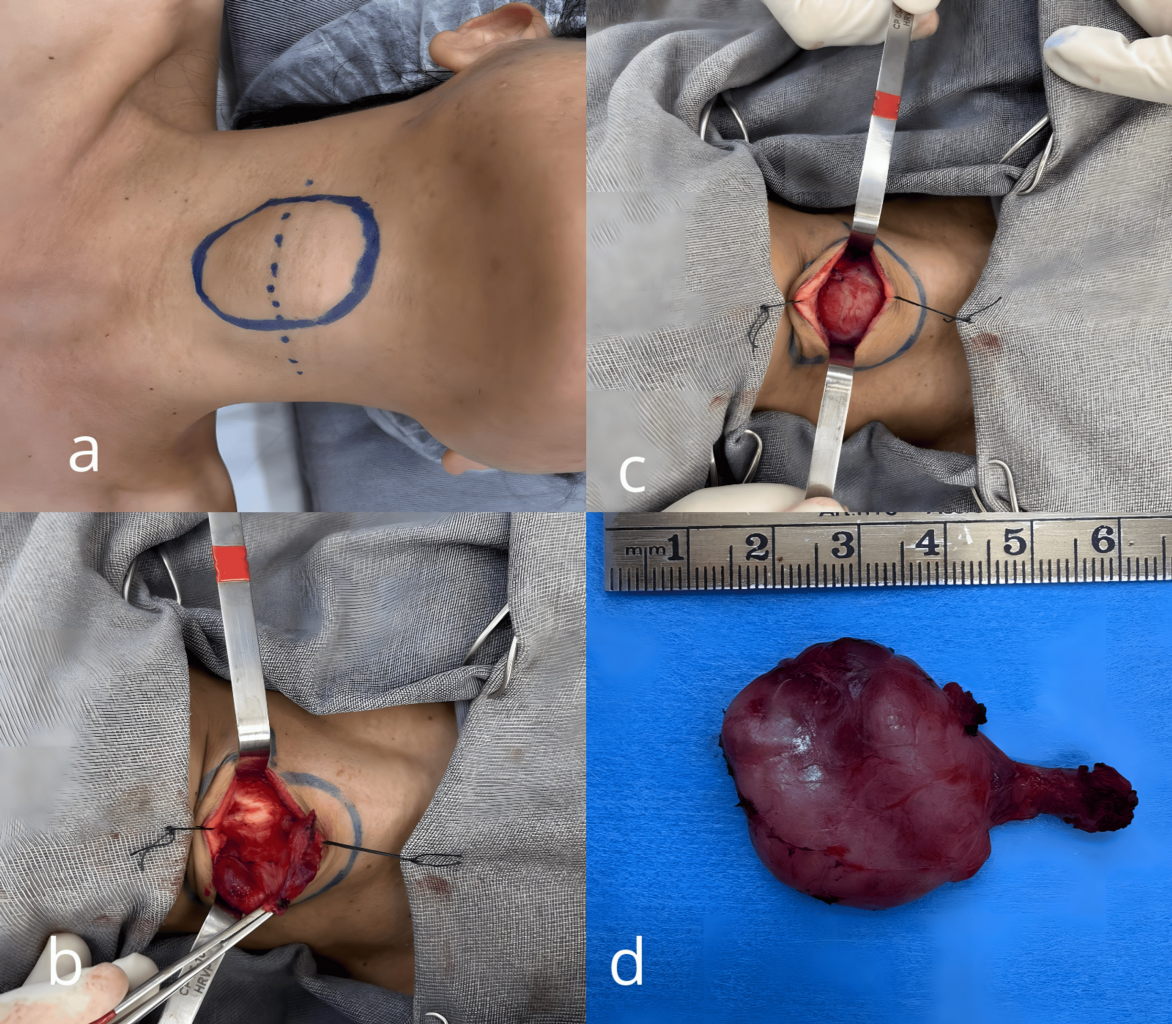
Surgical procedure in the cervical region Median transverse incision and dissection of the cervical ectopic nodule (a, b, c) and the excised specimen weighing 5.0 g.

**Figure 5:**
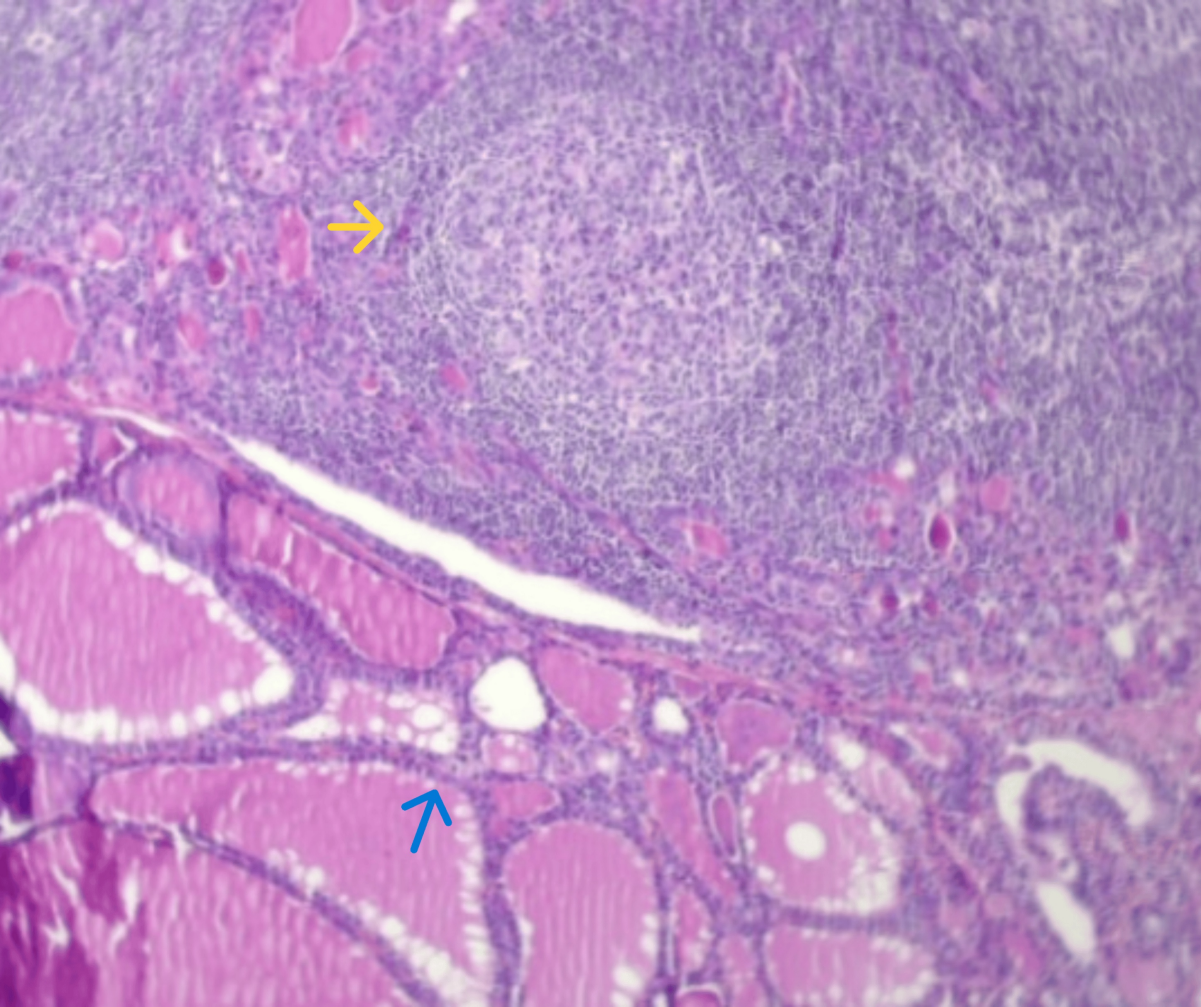
Histopathology of ectopic thyroid tissue Histopathological image, H&E stain (100x magnification). Note the dense lymphocytic infiltrate with formation of well-defined germinal centers (yellow arrow), consistent with chronic lymphocytic thyroiditis, and note the presence of mature thyroid follicles filled with colloid (blue arrow).

The surgical procedure was performed under general anesthesia and proceeded without complications. A median transverse incision was made over the lesion, followed by layered dissection to expose the nodule and allow its excision through meticulous dissection. Gross pathological examination revealed a nodule weighing 5.0 g and measuring 4.0 × 3.0 × 1.0 cm (Figure 4). Histopathological analysis demonstrated chronic lymphocytic thyroiditis with no evidence of malignancy (Figure 5). The patient had an uneventful postoperative course, with complete resolution of symptoms

## Discussion

The thyroid gland originates from a diverticulum formed by endodermal tissue during the third or fourth week of gestation, located on the median ventral wall of the pharynx. After the fourth week of gestation, a thickening of this tissue occurs at the midline of the primitive pharyngeal floor, forming the primitive thyroid. This structure penetrates the underlying mesenchyme, descending to occupy its definitive position in the lower neck, within the pretracheal region [9]. This process forms a narrow canal called the thyroglossal duct, which typically undergoes atrophy before the final formation of the thyroid gland. Disruptions in this process may result in the persistence of thyroid tissue along the migration pathway, leading to the development of ectopic thyroid tissue [9, 10]. Genetic factors also contribute to thyroid dysgenesis, with mutations in genes such as *NKX2-1*, *PAX8*, and *FOXE1 *being associated with abnormalities in thyroid development and migration [11].

The anatomical distribution of ectopic thyroid tissue varies widely, reflecting the embryological descent of the gland. The most common location is the lingual region, accounting for up to 90% of reported cases. Other midline locations include the sublingual, suprahyoid, and infrahyoid regions [12, 13]. Lateral cervical locations, although less frequent, have also been described, including the submandibular and parotid regions [14]. In rare instances, ectopic thyroid tissue has been identified in thoracic sites such as the mediastinum, heart, and lungs, as well as in abdominal locations, including the liver, pancreas, and ovaries [12]. Additionally, cases of dual ectopic thyroid, as observed in the present report, and even triple ectopic thyroid have been described, underscoring the complexity and variability of this condition [10].

In the present case, ectopic thyroid tissue was identified in two distinct locations: an expansile lesion at the base of the tongue and another in the right anterolateral cervical region at the level of the thyroid cartilage. This configuration represents a rare presentation involving multiple foci of ectopic thyroid tissue. The presence of ectopic thyroid tissue in a lateral cervical location, particularly in a paralaryngeal position as observed in this patient, is exceedingly uncommon [15, 16]. Although rare, such lateral cervical presentations have been reported and frequently raise differential diagnostic considerations, including metastatic papillary thyroid carcinoma, reactive lymphadenopathy, and parathyroid adenoma [15,17].

The diagnosis of ectopic thyroid may be suspected based on physical examination findings but requires imaging studies for confirmation. Ultrasonography is often the initial imaging modality of choice, as it allows adequate visualization of thyroid structures and adjacent anatomy and is widely available. CT and magnetic resonance imaging are useful for assessing the extent of the lesion and its potential compressive effects on surrounding structures [8]. Thyroid scintigraphy using technetium-99m or iodine-123 is considered the gold standard for confirming ectopic thyroid tissue, as it enables functional characterization of the gland [8,17] and helps exclude differential diagnoses such as thyroglossal duct cysts [12,15]. In the reported case, scintigraphy was not performed because the diagnosis of ectopic thyroid tissue had been previously established. However, due to the progressive increase in the size of the cervical nodule, a fine-needle aspiration biopsy was performed to exclude malignancy.

Management of ectopic thyroid depends on symptom severity and thyroid functional status. Asymptomatic patients with normal thyroid function may be managed conservatively with periodic follow-up. In cases of hypothyroidism, hormone replacement therapy with levothyroxine is indicated and may lead to a reduction in lesion size through negative feedback on TSH secretion [18]. When significant compressive symptoms, progressive lesion enlargement, or concern for malignant transformation are present, surgical intervention is warranted [16,19]. This recommendation is supported by evidence that ectopic thyroid tissue is more susceptible to hyperplasia, thyroid adenoma, thyroiditis, and carcinoma than orthotopic thyroid tissue [20].

As a case report, this study’s findings cannot be generalized, though its value lies in the rarity of the condition. The short follow-up limits definitive conclusions on long-term outcomes and potential tongue-base manifestations. Additionally, while radionuclide scintigraphy (gold standard) was not performed, the diagnosis was reliably confirmed by the clinical history and the high concordance between US, CT, FNAB, and definitive histopathology. The strengths of this study include the detailed report of a rare thyroid embryological migration failure, supported by robust multimodal documentation (imaging, surgical, and histopathological).

## Conclusions

Ectopic thyroid is a rare embryological anomaly resulting from the abnormal migration of the thyroid gland and can occur at multiple sites along the thyroglossal tract. The present case illustrates an uncommon presentation characterized by multiple foci of ectopic thyroid tissue in the absence of an orthotopic gland, causing significant symptoms for the patient. Diagnosis was established through ultrasonography, computed tomography, and videolaryngoscopy, which were essential examinations to guide surgical treatment, particularly due to the presence of compressive symptoms. It is worth noting that treatment must be individualized, considering the risks and benefits of surgical intervention, as well as the necessity for hormone replacement therapy in patients with hypothyroidism.
